# Enhanced Methods to Estimate the Efficiency of Magnetic Nanoparticles in Imaging

**DOI:** 10.3390/molecules22122204

**Published:** 2017-12-12

**Authors:** Ann M. Hirt, Monika Kumari, David Heinke, Alexander Kraupner

**Affiliations:** 1 Institute of Geophysics, Sonneggstrasse 5, ETH-Zürich, CH-8092 Zürich, Switzerland; monika.ethz@gmail.com; 2 nanoPET Pharma GmbH, Luisencarreé, Robert-Koch-Platz 4, D-10115 Berlin, Germany; david.heinke@nanopet.de (D.H.); alexander.kraupner@nanopet.de (A.K.)

**Keywords:** FeraSpin^TM^, MRI, MPI, magnetic properties, magnetic hysteresis, FORC, AC susceptibility, ZFC-FC magnetization

## Abstract

Magnetic resonance imaging (MRI) and magnetic particle imaging (MPI) are powerful methods in the early diagnosis of diseases. Both imaging techniques utilize magnetic nanoparticles that have high magnetic susceptibility, strong saturation magnetization, and no coercivity. FeraSpin^TM^ R and its fractionated products have been studied for their imaging performances; however, a detailed magnetic characterization in their immobilized state is still lacking. This is particularly important for applications in MPI that require fixation of magnetic nanoparticles with the target cells or tissues. We examine the magnetic properties of immobilized FeraSpin^TM^ R, its size fractions, and Resovist^®^, and use the findings to demonstrate which magnetic properties best predict performance. All samples show some degree of oxidation to hematite, and magnetic interaction between the particles, which impact negatively on image performance of the materials. MRI and MPI performance show a linear dependency on the slope of the magnetization curve, i.e., initial susceptibility, and average blocking temperature. The best performance of particles in immobilized state for MPI is found for particle sizes close to the boundary between superparamagnetic (SP) and magnetically ordered, in which only Néel relaxation is important. Initial susceptibility and bifurcation temperature are the best indicators to predict MRI and MPI performance.

## 1. Introduction

Iron oxide nanoparticles have attracted enormous interest in biomedical imaging, i.e., magnetic resonance imaging (MRI) and magnetic particle imaging (MPI) [1,2,3,4]. The tuning of iron oxide nanoparticles as a contrast agent for MRI and tracer in MPI can enhance the visibility of images. It is important, however, to understand how a material’s physical properties affect its magnetic properties. For example, it has been shown that superparamagnetic behavior and high magnetic moment are desirable for both MRI and MPI [5,6,7]. The magnetic properties of a material will depend upon the particles’ chemical composition, size, shape and whether there is any magnetic interaction amongst the particles [3,8]. Chemical composition will determine to a large extent the saturation magnetization of a material. For example, magnetite (Fe_3_O_4_) has a saturation magnetization (M_S_) of 92 Am^2^kg^−1^, whereas oxidizing the ferrous iron in its structure to ferric iron leads to the formation of either maghemite (γ-Fe_2_O_3_) with M_S_ of around 70 Am^2^kg^−1^, or hematite (α-Fe_2_O_3_) with M_S_ of around 0.4 Am^2^kg^−1^. Particle size plays an important role for a material’s coercivity and susceptibility, i.e., the initial rise in the magnetization curve in an applied field. How long a particle holds its magnetization is governed by the Néel-Arrhenius Equation (1):

(1) $$\tau =\tau_{o}\exp\frac{\mathrm{Kv}}{k_{B}T}=\tau_{o}\exp\frac{{\mathrm{vM}}_{s}H_{c}}{2k_{B}T}$$

where τ_o_ is known as the attempt time with a value on the order of 10^−9^ to 10^−10^ s, K is the anisotropy energy, v is particle volume, H_C_ is the coercivity, M_S_ is the saturation magnetization, k_B_ is Boltzmann’s constant, and T is temperature. If the magnetization decays within the time that a measurement is made, it is known as superparamagnetism. Superparamagnetic (SP) particles are homogeneously magnetized but have no coercivity; their magnetization curves can show saturation if their diameter is more than several nanometers. As the volume increases, the anisotropy energy becomes larger than the thermal energy and the particle will have a stable remanent magnetization. The particle is homogeneously magnetized, and this state is known as stable single domain (SD). If particle size further increases, it is no longer energetically favorable for the particle to be homogenously magnetized, and the particle organizes itself into domains to reduces the magnetization energy. As the number of domains increases within a particle, the coercivity decreases, so that a large particle would have no coercivity [9]. The magnetization within each domain organizes itself so that collectively the total energy is minimized, which reduces the magnetization in the absence of an applied field. This is known as multi-domain (MD) state.

For minerals with high M_S_, shape will influence the magnetic properties, because the magnetization prefers to lie along the longest direction of the particle. Therefore, magnetic properties, such as coercivity and in some cases susceptibility, will be strongest along the preferred direction of magnetization, and weakest normal to the preferred direction; the particles display anisotropic behavior. If particles are close enough so that they magnetically interact, interaction plays a role in modulating the effective magnetic particle size, the particle anisotropy and their uniform dispersion. This can lead to a decrease in M_S_ and coercivity, in which the decrease in coercivity is similar to what one would expect for a multi-domain particle.

In summary, the magnetic moments of SP particles can easily align within an applied field, which leads to a high initial susceptibility. Highest initial susceptibility is observed for particle, whose diameter is close to the boundary of SP and SD. The magnetic domains in MD particles will also be aligned in a field, which also leads to a high susceptibility, although not as high as in the SP state [10]. SD grains show the lowest susceptibility. Note that if interactions do not play a role, M_S_ would be the same for all particle sizes above a few nanometers.

It has been shown that SP particles contribute to enhanced visibility in MRI images, because they allow a decrease in T2 relaxation time of neighboring protons with a simultaneous increase in relaxivity R2 (1/T2) by introducing a magnetic field inhomogeneity in the target region, thus aggravating rapid dephasing of neighboring protons [11]. MPI on the other hand requires nanoparticles with a steep initial magnetization curve, which increases the harmonic spectrum [6]. The particle harmonic spectrum of a sample is influenced by the behavior of magnetic relaxation, i.e., Néel and Brownian relaxations, which is why SP particles are good in contrast agents. There are numerous studies underway to produce MNP with improved contrast properties for MRI, or tracer material for MPI [4,7,12,13,14]. MPI studies recommend that the MNP should be monodisperse with little anisotropy and a single core, and a particle size around 20–30 nm [7,15,16]. “Gold-standard” Resovist^®^ has been used as the standard for comparing the MPI signal strength in terms of harmonic spectra [14,17,18]. Since Resovist is no longer commercially available, efforts focus presently on synthesizing and testing new materials that will yield similar if not better, image quality. FeraSpin^TM^ R, manufactured by nanoPET Pharma GmbH (Berlin, Germany), is one example of such a product.

This study focuses on the magnetic properties of FeraSpin R and its fractions. FeraSpin R and Resovist are both superparamagnetic iron oxide—a mixture of magnetite and maghemite–nanoparticle suspensions coated with carboxydextran and having a hydrodynamic diameter of about 60 nm. Due to the shortening of the T2 relaxation time of neighboring protons, they are used as negative contrast agents in MRI. Since the particles are rapidly accumulated in the liver they are especially used for liver imaging. While Resovist is clinically approved for use in humans but is no longer commercially available, FeraSpin R is indicated for use in small animal imaging [19].

**Figure 1:**
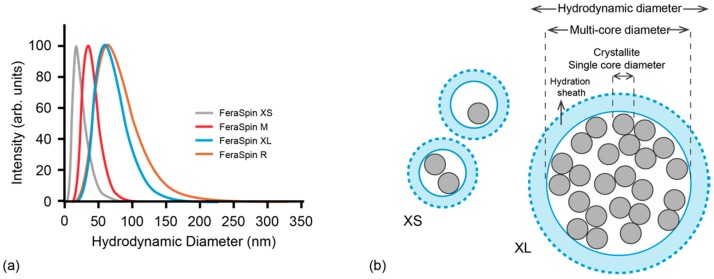
(**a**) Log-normal distributions of intensity-weighted hydrodynamic diameter of FeraSpin R, XS, M and XL, respectively as determined from DLS; (**b**) Schematic diagram illustrating the magnetic single-core-diameter (crystallite), of a multi-core diameter and the hydrodynamic diameter of sample FeraSpin XS versus FeraSpin XL.

FeraSpin R consists of elementary crystallites of iron-oxide, nominally γ-Fe_2_O_3_, with a diameter of 5–7 nm, according to which some of these crystallites aggregate to form particles with a larger diameter, such that there is a broader distribution due to these multi-cores [20] (Figure 1). The mean hydrodynamic diameter is 60 nm. A series of different multi-core size fractions have been isolated from FeraSpin R, namely, FeraSpin XS, FeraSpin M and FeraSpin XL, in which each fraction has a narrower multi-core size distribution than the parent material, and a mean hydrodynamic diameter of 15 nm, 35 nm, and 55 nm, respectively (Figure 1a). The magnetic core of FeraSpin XS has been shown from transmission electron microscopy (TEM) to be on the order of the elementary crystallites, with a mean of 5.8 nm, whereas FeraSpin L (not studied here) has larger aggregates with a mean magnetic core size of 33 nm [20]. Therefore, the average particle size of the fractions increases from XS to XL. Although several studies have been made on FeraSpin R and the FeraSpin series in relation to their performance in MPI [17,18,20,21,22,23], this study performs a more detailed magnetic characterization on the immobilized particles. The magnetic properties of the immobilized particles are compared to the magnetic properties and image performance of Resovist. Information on the static magnetic properties is essential, particularly for MPI applications that demands fixation of the MNP to cells and tissues. Results will help to distinguish the dependence of magnetic properties on particle size, and how this contributes to their final performance for MRI and MPI. We are specifically interested in whether the magnetic properties reflect the size of the elementary crystallites that constitutes a multi-core or particle aggregates. Here, aggregation can either be due to clustering of the elementary crystallites of a multi-core or clustering of the multi-core units. This would mean that the effective magnetic particle size can lie between the size of the crystallites to the size of the multi-core itself. Dipolar interaction between individual multi-core particles could lead to an even larger effective particle size, however coating should inhibit the magnetic interactions, so that the effective magnetic particle size is determined by the degree of aggregation of the crystallites (Figure 1b).

A series of different magnetic measurements have been made on immobilized samples to assess which magnetic parameters and methods are most suitable in predicting performance in MRI and MPI. The temperature dependence of low-field susceptibility is used to assess the chemical composition of the samples. First order reversal curves (FORC) [24,25,26] are used to define particle effective magnetic core-size distribution, fraction of SP and SD particles in a mixture [27], interaction among the individual crystallites [28,29], and the compositional purity [30,31]. We further test for interaction using FORC at 50 K and temperature dependent alternating current (AC) susceptibility. Induced magnetization is further monitored as a function of temperature after cooling in zero-field (ZFC) or in a field (FC) to define the average blocking temperature of the SP fraction. The magnetic properties are finally studied with respect to their performances in MRI and MPI. The results from this study are used to demonstrate the role of chemical composition, effective magnetic particle size and interactions on imaging performance. These findings should help manufacturers and end-users in tailoring the magnetic properties so that they lead to enhanced imaging.

## 2. Results

### 2.1. Magnetic Characterization

#### 2.1.1. Low-Field Susceptibility

**Figure 2:**
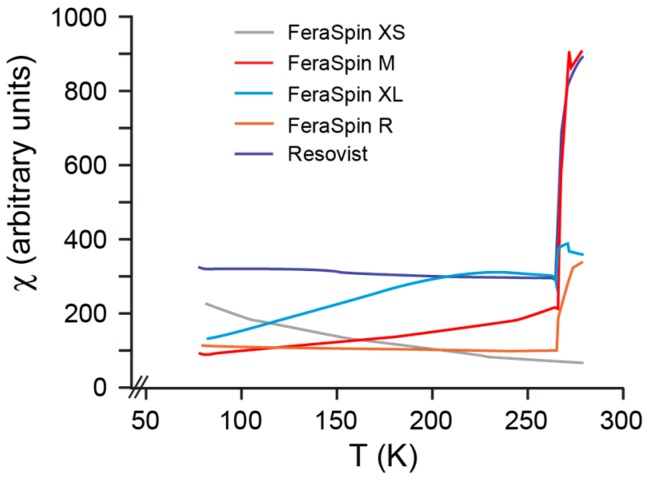
χ as a function of temperature illustrating the Morin transition.

Monitoring low-field, mass susceptibility (χ) as a function of low temperature shows that there is an abrupt loss at about 265 K in all samples (Figure 2) from the FeraSpin samples and Resovist. This susceptibility loss is characteristic for Morin transition in hematite (T_M_) [32,33], which indicates that all size fractions have undergone some degree of surface oxidation. The Morin transition is not readily apparent for FeraSpin XS, which can be suppressed due to high internal stress in very small particles [34,35]. The absence of a Verwey transition in all samples further indicates that the original Fe_3_O_4_ has experienced some degree of oxidation to γ-Fe_2_O_3_ [36].

#### 2.1.2. Induced Magnetization

**Figure 3:**
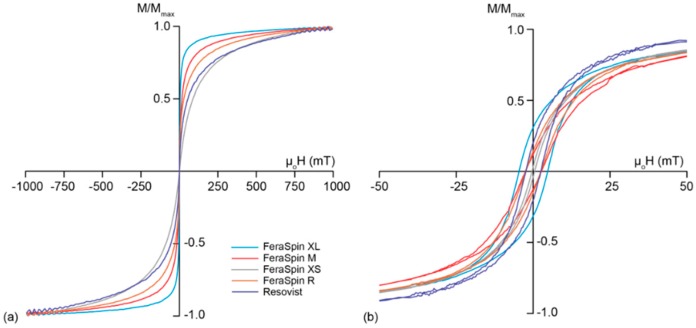
Magnetization curves for all samples with magnetization normalized by the maximum magnetization, (**a**) at 300 K, and (**b**) at 30 K.

The room temperature (RT) magnetic hysteresis loops of the particles in suspension are closed with virtually zero remanence for FeraSpin R, its multi-core size fractions (FeraSpi Series), and Resovist (Figure 3a), which signifies a predominance of SP particles. The magnetization curve of each sample shows a steep initial increase, but it should be noted that none of the samples reach magnetic saturation in a field of 1 T. This is due to the presence of α-Fe_2_O_3_ [37], although ultra-fine particles may also prevent saturation due to surface anisotropy [38]. Measurements were repeated on dried samples (data not shown), and there was no observable difference in the hysteresis loop, which supports that the samples are made up largely of particles with SP behavior, and the closed hysteresis loop is not due to physical (Brownian) rotation of the particles during measurement.

At 30 K, all samples have an open hysteresis loop (Figure 3b), indicating some portion of the particles undergo magnetic blocking at low temperature. FeraSpin XL with the largest hydrodynamic diameter has coercivity, µ_o_H_C_, of 11.7 mT. FeraSpin XS, the smallest multi-core fraction has a very thin magnetic loop at 30 K with µ_o_H_C_ = 1.1 mT. This suggests that an even lower temperature would be needed to block in the magnetic moment of all particles, and may reflect that crystallites show more limited interaction in the smallest particle fraction. FeraSpin M, FeraSpin R, and Resovist have a µ_o_H_C_ = 6.2 mT. The observed difference in µ_o_H_C_ suggests varying degree of aggregation among the elementary crystallites, whereby the degree of aggregation is related to the multi-core diameter.

#### 2.1.3. FORC Analysis

**Figure 4:**
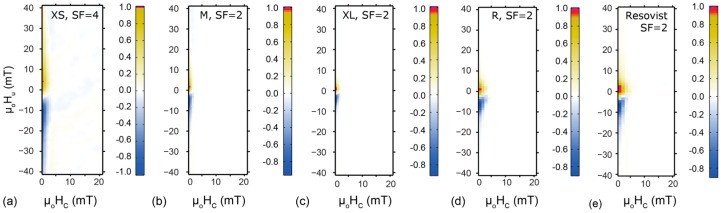
FORC distributions for (**a**–**c**) FeraSpin fractions; (**d**) FeraSpin R and (**e**) Resovist. Material name and smoothing factor SF are indicated at the top of each diagram.

FORC distributions for FeraSpin R, FeraSpin series and Resovist at room temperature are located close to the origin of the FORC diagram (Figure 4). All FORC diagrams show a low spread on the coercivity axis and a positive shift with respect to zero interaction field. This indicates the dominance of “non-interacting” SP particles [25,26]. FeraSpin XL shows the least spread of the FORC density distribution along the interaction axis, µ_o_H_b_, while FeraSpin XS has the largest. The difference suggests that there is more relaxation occurring during the measurement in FeraSpin XS compared to FeraSpin XL. The FeraSpin series suggests an increase in the positive shift in the peak interaction field with decreasing average particle size. FeraSpin R and Resovist have the broadest coercivity profiles extending up to 8 mT, indicating a larger fraction of particles with d ≥ 25 nm. Although the elementary crystallite size is the same in all samples, the broader coercivity distribution indicates that there is significant aggregation [12,22,39].

**Figure 5:**
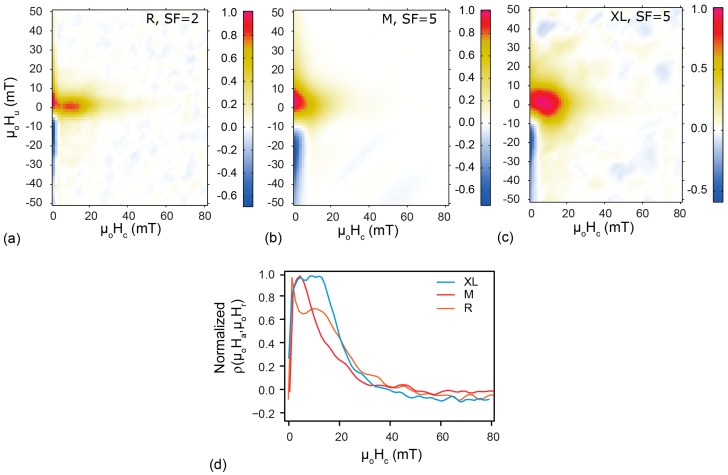
FORC distributions at 50 K for (**a**) FeraSpin R, and its fractions; (**b**) FeraSpin M; and (**c**) FeraSpin XL; (**d**) Corresponding coercivity spectra from FORC analysis.

Figure 5 displays FORC distributions of FeraSpin R, FeraSpin M and FeraSpin XL at 50 K. As temperature decreases the magnetic moments of all particles with an effective magnetic particle size in the SP range block in, i.e., become SD. The difference observed in the individual FORC diagrams at 50 K indicates aggregation among the elementary crystallites of 5–7 nm, which reflects the mean effective magnetic core-size for each sample. FeraSpin R (Figure 5a,d) has a bimodal FORC distribution, i.e., two distinct peaks in its coercivity distribution at about 2 mT and 11 mT. The peak coercivity at 2 mT is typical for very small magnetic particles that still are not completely blocked, while the higher coercivity comes from the particles that have undergone magnetic blocking. The fact that the coercivity profile persists until approximately 70 mT, may reflect blocked α-Fe_2_O_3_. The small spread along interaction axis accounts for little interaction among particles in FeraSpin R. FeraSpin M has a unimodal FORC distribution at 50 K with a spread in coercivity up to 50 mT, and a spread along interaction axis from −20 mT to 40 mT (Figure 5b,d). This suggests that there may be more interaction among the multi-cores that results in a larger effective magnetic particle-size. Note, however, the effective particles size is still SP at room temperature. FeraSpin XL shows a unimodal FORC distribution with a plateau in peak coercivity from 7 mT to 13 mT (Figure 5c,d). The coercivity distribution extends to higher fields, which probably arises from the α-Fe_2_O_3_. The FORC distribution is more contained at the origin, which suggests that the concentration of ordered particles, i.e., according to their effective magnetic size, is higher compared to the other samples. It also has a narrower spread at higher fields along µ_o_H_b_, which suggests little to no interaction among the multi-cores.

A semi-quantitative estimation for relative fractions of SP and SD particles in FeraSpin R, FeraSpin M and FeraSpin XL at 50 K was obtained by deconvolving the reversible and irreversible components of induced magnetization from the FORC data, assuming magnetite nanoparticles have uniaxial shape anisotropy [40] (Table 1). FeraSpin XL contains 75% of SD particles, while FeraSpin R and FeraSpin M both contain around 65–70% SD particles at 50 K.

#### 2.1.4. Zero-Field-Cooled and Field-Cooled Magnetization

**Figure 6:**
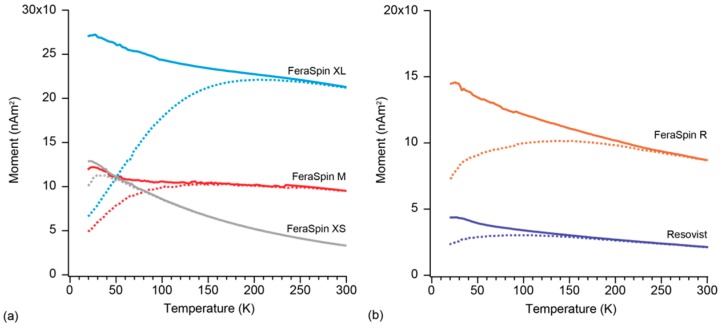
Temperature dependent ZFC (dotted)-FC (solid) magnetization curves for (**a**) FeraSpin fractions; and (**b**) FeraSpin R and Resovist.

The temperature dependence of magnetization in ZFC and FC curves shows that all samples exhibit superparamagnetism, in which the average blocking temperature (T_B_) can be defined from the temperature of peak magnetization in the ZFC curve (Figure 6). The temperature at which the first magnetic particles start to block is defined at the bifurcation point (T_S_) on the ZFC and FC curves (Table 1). All samples show a wide blocking spectrum except for FeraSpin XS, due to its narrow particle size distribution (Figure 1 and Figure 6). FeraSpin XL has T_S_ around room temperature, confirming the presence of larger crystallite aggregates that are blocked (Figure 6a). Resovist and FeraSpin R have similar values for T_S_ (Figure 6b). It should be noted that FeraSpin R has the broadest plateau at T_B_, which reflects a broad range of particle sizes, although the average effective magnetic particle size is nearly equal to that of Resovist.

#### 2.1.5. AC Susceptibility

**Figure 7:**
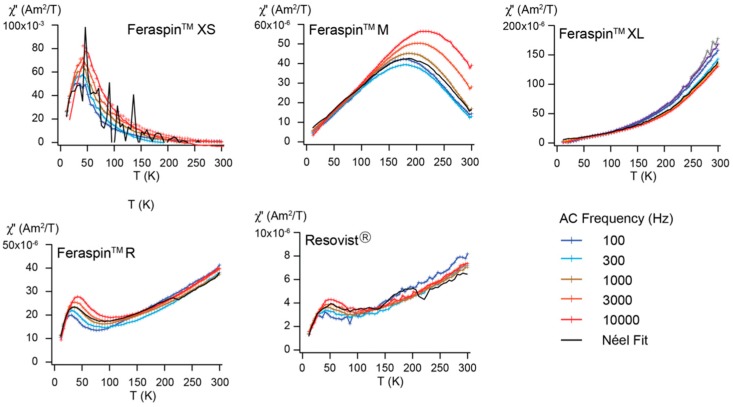
Out-of-phase contribution of the AC-susceptibility as a function of temperature at five frequencies compared with Néel relaxation.

AC susceptibility is used to determine the origin of thermal relaxation and magnetic interaction by decomposing it into its in-phase (χ′) and out-of-phase (χ″) susceptibilities (Figure 7). For all samples χ″ shows a good agreement with Néel relaxation, also known as the π/2-law [41,42], which indicates that thermal relaxation is solely due to the SP component in the samples. Each sample exhibits a unique χ″ susceptibility spectrum, where the temperature of peak χ″ is related to the average particle size under which the particles undergo blocking. Blocking temperature decreases from FeraSpin XL to FeraSpin M to FeraSpin XS. The absence of a true peak and the weak frequency dependency over the measured temperature range in χ″ in FeraSpin XL, indicates that the average T_B_ is close to room temperature. FeraSpin R and Resovist carry two T_B_, one at approximately 40 K representing contribution from fine particles and the second around 300 K that is indicative of larger particles. This bimodal distribution has been modeled from measurements of magnetization versus frequency [43]. Because FeraSpin XS and FeraSpin M display χ″ susceptibility characteristic for a unimodal size distribution, the Néel-Brown equation was used to evaluate the pre-factor or attempt time, τ_o_. In this case τ_o_ = 9.9 × 10^−132^ s for FeraSpin XS. This has no physical meaning, and is interpreted as reflecting interaction in the particle system. FeraSpin M has τ_o_ = 1.1 × 10^−14^ s, which is slightly high with respect to the empirically defined range of 10^−8^ to 10^−11^ s [44,45], which suggests that interaction may be influencing the magnetic properties to some extent.

### 2.2. Imaging Performance

The contrast efficacy of magnetic nanoparticles in MRI is a function of their spin-lattice relaxivity (R_1_) and spin-spin relaxivity (R_2_). Iron-oxide nanoparticles in the SP size range indirectly cause a rise in the image contrast by altering the relaxation times of neighboring protons, and this is characterized by their relaxivities. T2-weighted image performance is expressed in terms of R_1_, R_2_ and R_2_/R_1_ (Table 2) [46]. FeraSpin XL has the highest negative contrast efficacy in MRI (high R2/R1 value) and the fraction FeraSpin XS the least.

MPI performance can be described by the richness of the harmonic spectrum of the sample, i.e., high spectral amplitude and a less decay at high harmonic number. Here, the relative ranking of MPI signal strength is based on the harmonic spectrum given in the literature [21,22,47] for FeraSpin fractions and Resovist (Table 2) with 1 indicating the highest harmonic amplitudes and 4 the lowest. The MPI performance of FeraSpin R and Resovist is comparable due to their similar harmonic spectra [18,19]. The largest particle size fraction has the best performance in suspension, but the FeraSpin M particle size is best when immobilized. In both the cases FeraSpin XS shows the least suitability for MPI.

## 3. Discussion

The hysteresis curves for each sample are identical for suspended and immobilized samples. This indicates that immobilization of these samples through solvent evaporation has no effect on their chemical and magnetic properties. All samples are dominated by SP behavior at room temperature, despite the difference in the amount of magnetic aggregation between the elementary crystallites. They are, however, no longer chemically pure Fe_3_O_4_, but a mixture with γ-Fe_2_O_3_ and α-Fe_2_O_3_, as seen from the Morin transition (Figure 2). A clear relationship can be seen in the lack of magnetic saturation and T_B_ (Figure 2, Table 1). As discussed above, very fine particles that are on the order of several nanometers may not magnetically saturate, but the presence of the Morin transition, suggests that α-Fe_2_O_3_, which has a high coercivity, is responsible for the high field contribution to the magnetization. FeraSpin XS, for example, has the smallest T_B_, but also the largest contribution of the high coercivity α-Fe_2_O_3_ as seen in the magnetization curves. FeraSpin XL on the other hand has the least contribution from α-Fe_2_O_3_ and the largest TB.

Although oxidation will reduce the effective core of Fe_3_O_4_ crystallites, FeraSpin XL has the largest average effective magnetic particles size, based on the average blocking temperature, followed by FeraSpin M, FeraSpin R and Resovist, which have a similar average effective magnetic size, and FeraSpin XS as the smallest particles. This means that although all samples have crystallites of similar size, the effective magnetic particles size follows the same trend as the physical particle size in the FeraSpin fractions [21]. The effective magnetic particle size should be reflected by T_S_ in the ZFC-FC magnetization curves and T_B_ in both the ZFC magnetization and AC susceptibility curves, and we find the lowest T_S_ and T_B_ for FeraSpin XS and the highest temperatures for FeraSpin XL. It is interesting to note that FeraSpin R has T_B_ similar to FeraSpin M, but T_S_ occurs at a higher temperature. This indicates that the average effective magnetic particle size is similar, but FeraSpin R has a broader particle size distribution. We also find that FeraSpin R and Resovist have a bimodal mean effective magnetic particle size distribution, with one part similar to FeraSpin XS and the other more similar to FeraSpin XL [20]. FORC analysis at 30 K, which is below TB for all samples except FeraSpin XS, shows that FeraSpin R and FeraSpin XL have limited interaction between aggregations within the multi-core, whereas FeraSpin M has more particle interaction. This was further verified from the predicted τo for FeraSpin M, although the amount of interaction cannot be large, because χ″ conforms to Néel relaxation (Figure 7).

**Figure 8:**
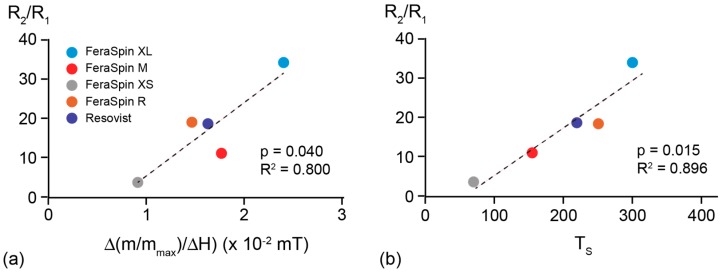
Relaxivity ratio as a function of (**a**) the initial slope of the moment normalized magnetization curve, up to 20 mT; and (**b**) average T_S_. In both cases the probability value is <0.05, suggesting >95% significance of the relationship between the variables in the linear regression model of the data set. R-squared is a statistical measure of how close the data are to the fitted regression line (dashed line).

If we compare the MRI performance with the magnetic results, the relaxivity ratio displays a linear dependency on susceptibility, defined from the initial slope of the magnetization curve between 0 to 20 mT (Figure 8a), and bifurcation temperature T_S_ (Figure 8b). These correlations show that MRI performance is controlled by the average effective particle size of the magnetic nanoparticles. Ideally particles should have a high susceptibility, which has been noted in other studies [6,7,21,23]. In practice this means that the particles should be slightly under the boundary between SP and SD particle size, because these have the highest susceptibility [48]. Deviation from the linear relationship, however, can arise from (i) inter-particle interactions and agglomeration, which would lead to a larger effective magnetic particle size, (ii) changes in composition, such as oxidation to a less magnetic phase, or (iii) particle shape.

For MPI performance there is a distinct difference whether the particles are in suspension or fixed. In the suspended samples, best performance was found for FeraSpin XL followed by FeraSpin R, M and XS. In this case, the samples that are close to the SP-SD boundary show the best performance. Because the particles of FeraSpin R and Resovist can be described by a bimodal mean effective magnetic particle size distribution and the small size fraction has only a small contribution to the overall magnetic particle spectroscopy (MPS) signal, the MPS performance is in comparison to XL, which consists of a large particle size fraction, decreased. FeraSpin XS consists solely of small single-core crystallites, which explains it having the least MPS signal.

In clinical applications, e.g., stem cell labeling [49], the particles’ mobility will be highly restricted, e.g., due to protein adsorption or a higher viscosity, resulting in a dynamic magnetic behavior more comparable to a fixed/immobilized particle. It is interesting to note that the harmonic spectrum was broader for the fixed FeraSpin M fraction compared to the fixed FeraSpin XL. Fixing the particles affects their magnetic behavior by suppressing the Brownian motion, thus only Néel relaxation can occur. Because FeraSpin M show no significant differences between the dispersed and immobilized state we can conclude that for FeraSpin M the internal Néel relaxation dominates also in the suspension, while for XL the Brownian motion dominates. It is possible that because FeraSpin XL is very close to being blocked, i.e., on the SP-SD boundary, that fixation causes the particle to behave more SD-like, hence lowering their initial susceptibility. This is in agreement with earlier studies on Resovist [14] and other tailored nanoparticle tracers [7].

In summary, average T_S_, obtained from ZFC, and initial susceptibility are both useful in predicting performance for MRI and MPI. T_S_ may be better suited when the particles have a broad size distribution. In terms of understanding magnetic properties, magnetization curves (hysteresis loops) provide evidence if there is a difference in magnetic composition when comparing samples. In our case, the slope of the magnetization curve in high field reflects the amount of oxidation of the sample from Fe_3_O_4_ to α-Fe_2_O. For this reason initial susceptibility reflects not only particle size, but also composition. The FORC diagrams showed that all samples were SP at room temperature, but that the magnetic core size varied as seen from the different coercivity spectra at 30 K. Because the samples remain SP even though there is aggregation of the crystallite, suggests that the size does not exceed the SP-SD boundary. The FORC results at low temperature also suggest that the crystallites self-assemble, such that the lattice structure is aligned, because, except for FeraSpin XS, interaction in the core is not significant. In this case, the aggregated particle magnetic behavior is similar to a single particle with equivalent size [50] Although both ZFC magnetization and AC susceptibility provide information on average blocking temperature, AC susceptibility was able to distinguish bimodal particle size distributions in FeraSpin R and Resovist in contrast to unimodal distributions in FeraSpin M and XS.

## 4. Materials and Methods

The intensity-weighted mean hydrodynamic diameter was determined by dynamic light scattering (DLS), by assuming a monomodal and log-normal size distribution. Note, that although FeraSpin R contains a fraction with single core particles, as found in FeraSpin XS, in addition to another fraction with aggregates of different sizes, DLS cannot resolve this bimodality and instead provides the mean size.

All magnetic measurements were performed on dried samples immobilized in a sealed capillary tube. In addition, hysteresis loops were also measured on suspended samples in the same manner as the dry samples. Low-field susceptibility was measured as a function of low temperature on an AGICO KLY2 susceptibility bridge, equipped with a cryostat, in a field strength 200 Am^−1^. A Princeton Measurement Corporation (PCM, now part of Lake Shore Cryogenics, Westerville, OH, USA) vibrating sample magnetometer (VSM, model 3900) at the Laboratory of Natural Magnetism, ETH-Zurich was used to measure induced magnetization as a function of field, FORC curves, and ZFC-FC. Multiple segment hysteresis loops were measured with a field of ±1 T and 100 ms averaging time.

First-order reversal curves (FORC) is a technique that uses a series of partial hysteresis loops to construct the coercivity distribution within a sample, and whether particle interactions occurs [24,25,26]. The sample is saturated initially in a large field, and then has its magnetization measured incrementally from a reversed field (H_a_) back to saturation. Each FORC with its reverse field is described by its magnetization M(H_a_, H_b_), in which H_b_ > H_a_, and the FORC distribution is obtained by a mixed second derivative:

(2) $$\rho \left(H_{a},H_{b}\right)=-\frac{\delta^{2}M\left(H_{a},H_{b}\right)}{2{\delta H}_{a}{\delta H}_{b}}$$

The FORC diagram is obtained by transforming the coordinate system from (H_a_, H_b_) to (H_c_, H_b_), where H_c_ describes the distribution of coercive force, H_c_ = (H_b_ − H_a_)/2, and H_u_ describes the distribution of local interaction fields, H_u_ = (H_b_ + H_a_)/2. A series of 140 FORC were made using 1.2 mT field increment and 100 ms averaging time; data were processed with Winklhofer MATLAB code [51]. The reversible and irreversible changes in magnetization have been isolated using the procedure outlined in [52]. Extracting the SP content is based on the method used in [40,52]. This method assumes that for non-interacting SD particles the reversible contribution makes up 50% of the magnetization, which is the case for Fe_3_O_4_ or γ-Fe_2_O_3_, dominated by shape anisotropy. Peak susceptibility, ∂M/∂H, is then used to gain a semi-quantitative estimate of the SP fraction.

Low temperature measurements were achieved using a cryostat on the VSM. For the zero-field-cooled and field-cooled (ZFC-FC) measurements demagnetized samples were initially cooled from 300 K to 20 K in absence of H, and then induced magnetization was measured as a function of temperature with a weak field of 10 mT with a 2 K temperature increment back to 300 K. Similarly, FC data were obtained by cooling the sample from RT to 20 K in a 1 T applied field. The field was then removed and a field of 10 mT was applied during warming. AC-susceptibility was measured as a function of temperature on a Quantum Design Physical Properties Measurement System (PPMS) at the Institute of Metal Research, ETH Zurich in five frequencies of 100, 300, 1000, 3000 and 10,000 Hz. Measurements were made between 10 K and 300 K with a measurement interval of 5 K. The π/2-Law, which relates the in-phase susceptibility to the quadrature susceptibility in the case of Néel relaxation, is defined by using χ′ that has been measured in two different frequencies, v_LF_ and ν_HF_, where in which ν_HF_ > ν_LF_, as:

(3) $$\mathrm{Néel}\;\mathrm{Relaxation}=-\frac{\pi }{2}\left[\frac{\chi_{\mathrm{LF}}^{\prime }-\chi_{\mathrm{HF}}^{\prime }}{\ln\left(\nu_{\mathrm{LF}}\right)-\ln\left(\nu_{\mathrm{HF}}\right)}\right]$$

Néel relaxation was tested for the differences in the quadrature susceptibility (χ″) between ν_HF_ = 3 kHz and ν_HF_ = 100 Hz for all FeraSpin samples, and with ν_HF_ = 3 kHz and ν_HF_ = 300 Hz for Resovist.

## 5. Conclusions

The FeraSpin samples are Fe_3_O_4_/γ-Fe_2_O_3_ with some degree of aggregation and oxidation to hematite. Both affect only the effective magnetic particle size, as seen from TB. MRI performance can be assessed on a first order from the magnetization curve and TS, whereby the larger the bulk particle size within SP range the better the performance. This holds because magnetite particles on the SP-SD boundary have the highest susceptibility and can easily align in a field. Bifurcation point may be the better parameter for judging performance, because it is less sensitive to changes in composition as is the case of initial susceptibility. The same is valid for MPI performance on SP particles in suspension. Fixing the particles, however, shows that particles close to magnetic ordering, i.e., SD behavior, did not perform as well, because these are dominated by Brownian motion rather than Néel relaxation. Thus, fixation may lead to a state in which the particles undergo magnetic ordering and therefore cannot respond as easily to an applied field. Although a broader study of different magnetic particles would aid in verifying the linear relationship of imaging performance with T_S_, our results support that the bifurcation point could be an easy and quick method in quantifying or assessing the efficiency of any new materials for imaging.

## Figures and Tables

**Table 1:** Semi-quantitative estimation of SP and SD fraction with the corresponding T_S_ and T_B_.

Sample Name	% Fraction of SP Particles (50 K)	% Fraction of SD Particles (50 K)	T_S_ (K)	T_B_ (K)
FeraSpin XS	-	-	70	30
FeraSpin M	31	69	155	140
FeraSpin XL	25	75	ca. 300	210
FeraSpin R	34	66	250	150
Resovist	-	-	220	90

**Table 2:** Performance strength for MRI and MPI.

Sample Name	R_1_^1^	R_2_^1^	R_2_/R_1_^1^	MPI (Suspension)	MPI (Fixed)
FeraSpin XS	13.0	49	3.8	4	4
FeraSpin M	9.9	117	11.2	3	1
FeraSpin XL	7.9	270	34.2	1	2
FeraSpin R	10.0	185	18.5	2 ^2^	3
Resovist	9.3	174	18.7	2 ^2^	NA

^1^ MRI measurements were made at 1.4 T and 300 K. ^2^ MPI performance by suspended FeraSpin R and Resovist exhibit same harmonic spectrum [22].
